# Multigenerational impacts of bile exposure are mediated by TGR5 signaling pathways

**DOI:** 10.1038/s41598-018-34863-0

**Published:** 2018-11-15

**Authors:** Marine Baptissart, Lauriane Sèdes, Hélène Holota, Laura Thirouard, Emmanuelle Martinot, Angélique de Haze, Betty Rouaisnel, Françoise Caira, Claude Beaudoin, David H. Volle

**Affiliations:** INSERM U1103, Université Clermont Auvergne, CNRS UMR-6293, GReD, F-63000 Clermont–Ferrand, France

## Abstract

Besides their well-known roles in digestion and fat solubilization, bile acids (BAs) have been described as signaling molecules activating the nuclear receptor Farnesoid-X-receptor (FXRα) or the G-protein-coupled bile acid receptor-1 (GPBAR-1 or TGR5). In previous reports, we showed that BAs decrease male fertility due to abnormalities of the germ cell lineage dependent on Tgr5 signaling pathways. In the presentstudy, we tested whether BA exposure could impact germ cell DNA integrity leading to potential implications for progeny. For that purpose, adult F0 male mice were fed a diet supplemented with cholic acid (CA) or the corresponding control diet during 3.5 months prior mating. F1 progeny from CA exposed founders showed higher perinatal lethality, impaired BA homeostasis and reduced postnatal growth, as well as altered glucose metabolism in later life. The majority of these phenotypic traits were maintained up to the F2 generation. In F0 sperm cells, differential DNA methylation associated with CA exposure may contribute to the initial programming of developmental and metabolic defects observed in F1 and F2 offspring. Tgr5 knock-out mice combined with *in vitro* strategies defined the critical role of paternal Tgr5 dependent pathways in the multigenerational impacts of ancestral CA exposure.

## Introduction

The last decade has presented evidences that offspring development, behavior and physiology can be determined by parental experiences^1^. In particular, rodent models of paternal exposures to malnutrition, stress or environmental contaminants have been associated with higher susceptibility to metabolic diseases across several generations^2,3^. Deeper explorations identified altered epigenetic patterns in mature sperm as strong candidates for multigenerational programming of diseases^3–6^.

Indeed, all along their differentiation male germ cells undergo dynamic epigenetic changes particularly sensitive to environmental stressors. Germ cell genome is progressively methylated to reach high level of DNA methylation in mature sperm^7^. This process relies on the activity of the *de novo* DNA methyltransferase enzymes DNMT3a and DNMT3b that are highly expressed within the germline. After fertilization, most alterations of DNA methylation levels that may occur during spermatogenesis are erased to allow *de novo* programming of the embryo in both somatic and germ cell lineages. However, recent findings suggest that certain alterations can escape a functional erasure and be disseminated over subsequent generations contributing to epigenetically inherited traits^8^.

Beside DNA methylation, incorrect histone patterns have been shown to mediate the effects of parental exposures on offspring health^9,10^. If most histones are replaced by protamines during spermiogenesis, 1% to 4% of the mature sperm genome remains associated with nucleosomes. Maintained on the paternal genome after fertilization, the nature of histone modifications and their position on the genome will initiate the proper transcriptional program required for early development of the embryo. The histones inherited from the spermatozoa will also guide the processes of demethylation-methylation of the future zygote genome in both somatic and primordial germ cells. Independently or in interaction with DNA methylation, retained histones contained in the mature spermatozoa are potential messengers of the effects of parental exposures on offspring physiology.

It has been established that bile acids (BAs) act as signaling molecules and regulate many physiological functions, such as lipid, glucose and energy metabolisms *via* the modulation of their two main receptors: the G-protein-coupled bile acid receptor-1 (GPBAR-1, TGR5) and the nuclear receptor Farnesoid-X-receptor alpha (FXRα, NR1H4)^11–13^. In a recent report, we showed that chronic exposure to a diet supplemented with 0.5% cholic acid (CA-diet) decreases sperm count and reduces male fertility. Testicular defects associated with the phenotype are dependent upon the activation of the membrane BA receptor Tgr5 within the germ cell lineage^14^.

Male germ cells contain fundamental epigenetic and genetic informations, which constitute the molecular basis of the paternal contribution to the next generations. Considering our previous finding, the question arises of whether BAs could impact germ cell integrity and contribute to the programming of phenotypic traits across multiple generations.

The present study shows evidence of altered DNA methylation levels in sperm cells from wild-type males exposed to CA-diet (F0^CA;+/+^). These differential methylation patterns are associated with high perinatal mortality in the first generation of pups originating from F0^CA;+/+^ founders (F1^CA;+/+^). In addition, surviving F1^CA;+/+^ offspring showed metabolic abnormalities, such as altered BA homeostasis and glucose intolerance at adult age. The F1^CA;+/+^ males are in turn able to transmit most of these phenotypic traits to the subsequent F2^CA;+/+^ generation. Interestingly, none of these developmental or metabolic abnormalities was observed in progeny born from CA exposed founders deficient for the gene encoding Tgr5 (F0^CA;−/−^). All together these data show that Tgr5 signaling pathways are critical to initiate multigenerational programming of phenotypic traits after paternal exposure to CA.

## Results

### Paternal exposure to cholic acid affects developmental and metabolic physiology across 2 generations of offspring

We previously demonstrate that pathological level of bile acids alter male fertility *via* Tgr5 in response to adult exposure to a diet supplemented with 0.5% of cholic acid (CA-diet)^14^. Remaining questions are to define if CA-exposure can impair germ cell integrity and if it could contribute to multigenerational programing of diseases in progenies.

**Figure 1:**
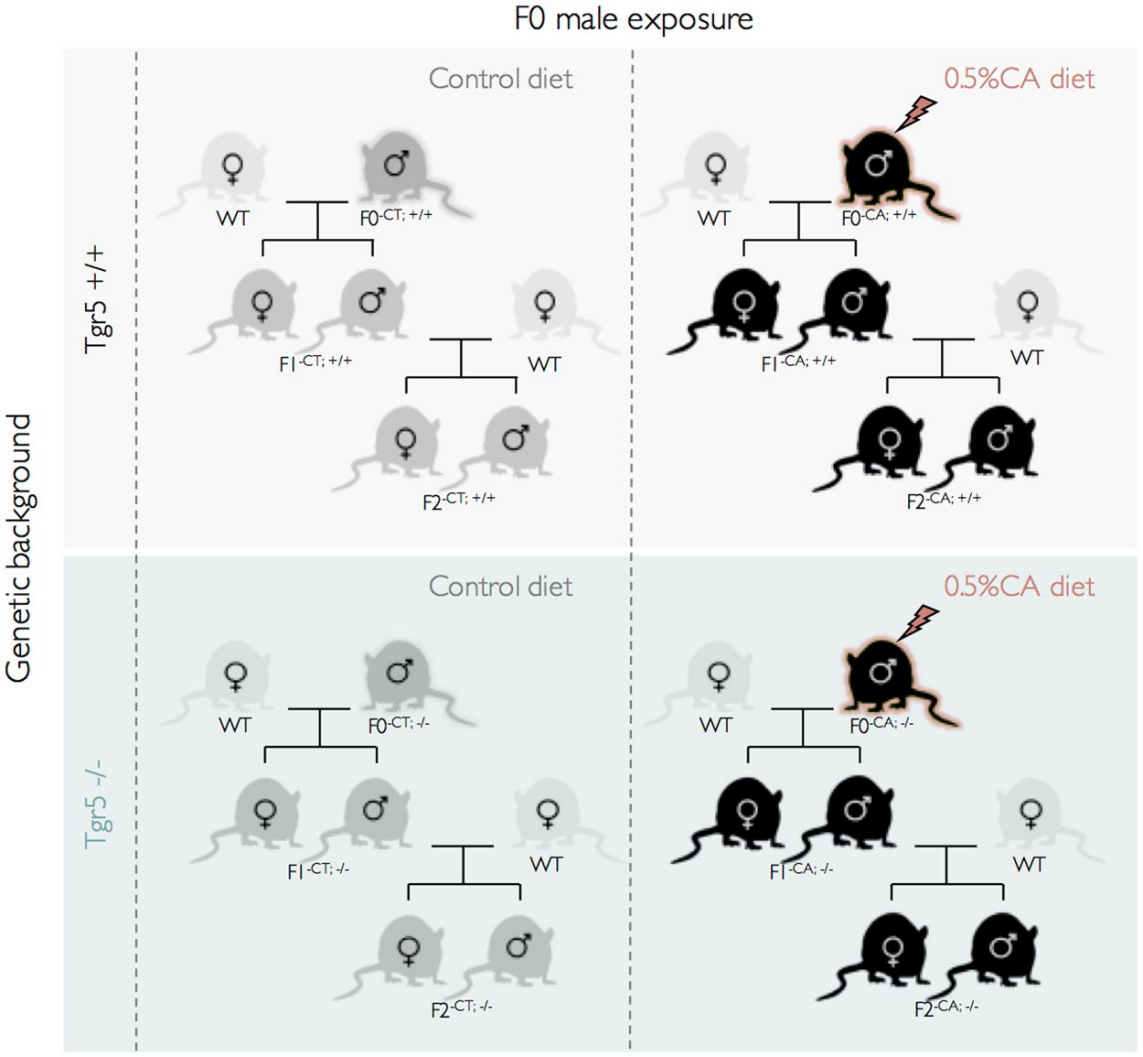
Experimental approach to analyze the multi-generational impacts of the paternal exposure to CA-diet. Adult wild type (F0^+/+^) and *Tgr5* knock-out (F0^−/−^) male founders were exposed to a control diet (CT) or the corresponding diet supplemented with 0.5%-CA (CA). After 3.5 months of exposure, males of each group were bred with unexposed C57BL6J females generating the experimental F1 offspring. These F1 males were then bred with unexposed C57BL6J females generating the experimental F2 offspring.

**Figure 2:**
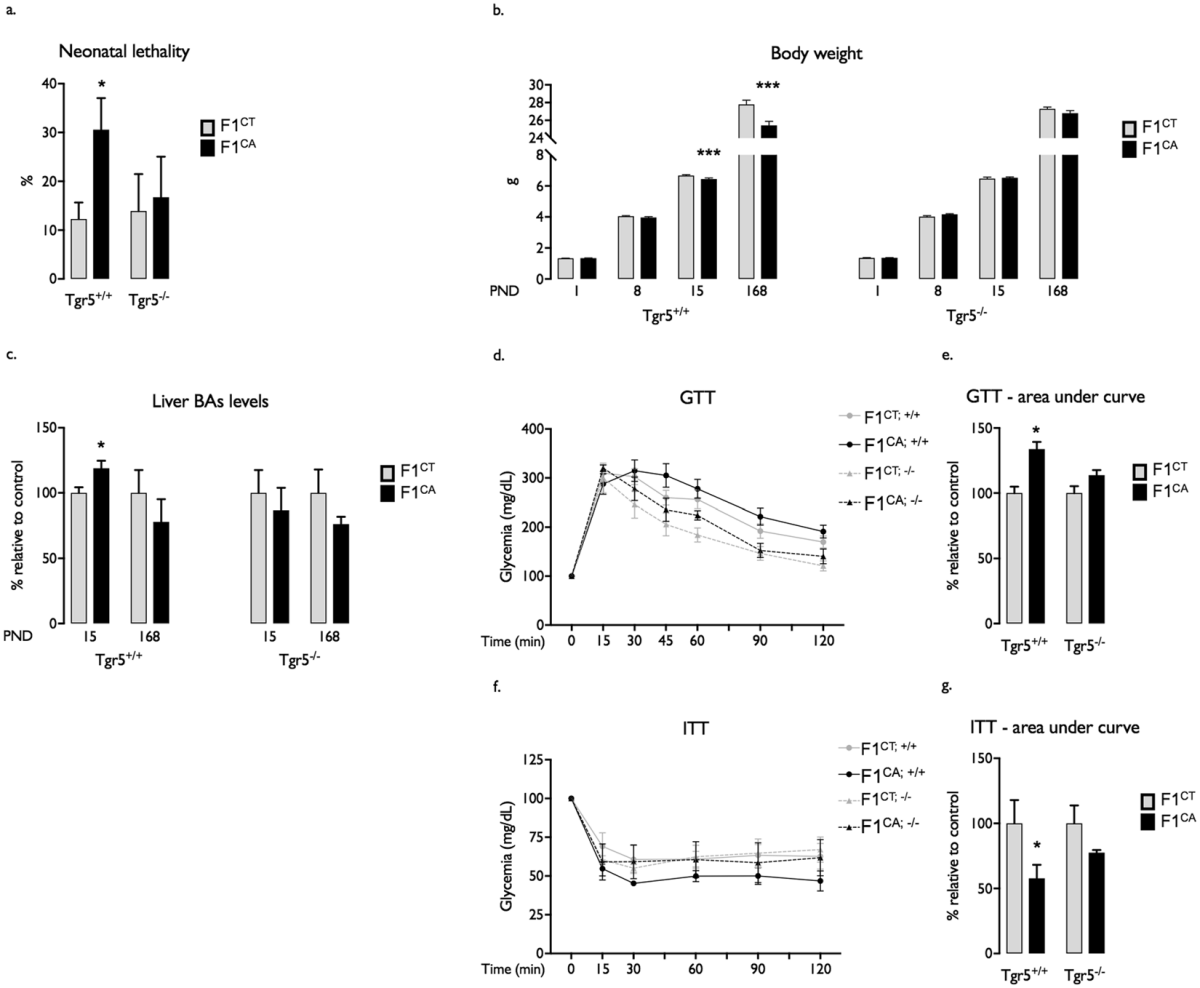
Paternal exposure to CA-diet impacts F1 offspring. (**a**) Percentage of neonatal mortality in F1 litters obtained from F0^+/+^ or F0^−/−^ fathers fed 3.5 months with CT or CA diet. (**b**) Body weight of F1^CT;+/+^, F1^CT;−/−^, F1^CA;+/+^ and F1^CA;−/−^mice at 1, 8, 15 and 168 postnatal days. (**c**) Hepatic bile acid levels in 15-days old and adult F1^CT;+/+^, F1^CT;−/−^, F1^CA;+/+^ and F1^CA;−/−^ mice. (**d**) GTT in adult F1^CT;+/+^, F1^CT;−/−^, F1^CA;+/+^ and F1^CA;−/−^ mice. (**e**) Area under the curve for GTT. (**f**) ITT in adult F1^CT;+/+^, F1^CT;−/−^, F1 ^CA;+/+^ and F1^CA;−/−^ mice. (**g**) Area under the curve for ITT. Data are expressed as means +/− SEM. In all panels for each group, *n* = 10–20 males from 3 to 5 independent experiments; *significance; *p* < 0.05.

For that purpose, adult wild type (F0^+/+^) and *Tgr5* knock-out (F0^−/−^) male founders were exposed to a control diet (CT) or the corresponding diet supplemented with 0.5%-CA (CA) as previously reported^14^. After 3.5 months of exposure, males of each group were bred with unexposed C57BL6/J females generating the experimental F1 offspring (Fig. 1). Consistently with previous report^14^, 20% of the males exposed to CA-diet (F0^CA;+/+^) were sterile (Supplemental 1a). Moreover, 30% of F1^CA;+/+^ progeny, originating from fertile F0^CA;+/+^ male founders, died in the first 2 weeks of postnatal life (Fig. 2a). Surviving F1^CA;+/+^ pups showed lower body weight at post-natal day 15 (PND15) compared with F1^CT;+/+^ originating from F0^CT;+/+^ founders (Fig. 2b). This difference was maintained up to adulthood (24 weeks old) (Fig. 2b). Males and females were affected in a similar way (Supplemental 1b,c). Interestingly, no impact of paternal CA exposure was observed in litters originating from Tgr5 knock-out mice (F1^CA;−/−^) compared to F1^CT;−/−^ mice (Fig. 2a,b).

Further analysis at PND15 showed higher hepatic BA levels in F1^CA;+/+^ group compared to F1^CT; +/+^ (Fig. 2c). According to previous studies^15^, altered BA homeostasis might contribute to postnatal lethality of F1^CA;+/+^. Again, no change in BA concentrations was observed in offspring originating from Tgr5 knock-out (F0^CA;−/−^) mice exposed to CA diet compared to control F0^CT;−/−^ animals (Fig. 2c).

To determine whether early BA increase in F1^CA;+/+^ offspring was maintained and potentially associated with other metabolic defects at adulthood, we extended our analysis and performed glucose and insulin tolerance tests (GTT, ITT) (Fig. 2d,g). No change in hepatic concentrations of BAs was detected in adult F1^CA;+/+^ males compared to F1^CT;+/+^ males (Fig. 2c). However, adult F1^CA;+/+^ males showed difficulties to restore glucose levels during GTT compared to their respective controls (Fig. 2d,e). Consistently with an impaired glucose tolerance, the F1^CA;+/+^ males showed lower fasting insulin concentrations before and along the GTT protocol (Supplemental 1d) and lower abilities to normalize glycemia when challenged with exogenous insulin (ITT) compared to F1^CT;+/+^ males (Fig. 2f,g). Of note, no significant alterations of glucose metabolism due to paternal CA exposure were observed in F1^CA;−/−^ offspring (Fig. 2d–g).

To gain functional insight into such altered metabolic signature, F1^CA;+/+^ and F1^CT;+/+^ male mice aged of 5 weeks were challenged with a high-fat diet. After 35 days of treatment, F1^CA;+/+^ males showed a higher body weight gain compared to F1^CT;+/+^ (Supplemental Fig. 1e,f).

All together, these results demonstrate that paternal CA exposure increases susceptibility to developmental defects and metabolic disorders.

### Paternal CA-exposure of F0 males results in postnatal abnormalities up to the F2 generation

Several evidences in the literature demonstrated that parental exposures could determine offspring physiology across several generations. After establishing perinatal lethality and impaired metabolism in F1^CA;+/+^ males, we raised the question of whether F2 offspring reproduced part of these phenotypic traits in the absence of further exposure. For that purpose, F1^+/+^ males were mated to unexposed C57BL/6 females generating F2^CA;+/+^ or F2^CT;+/+^ offspring (Fig. 1).

**Figure 3:**
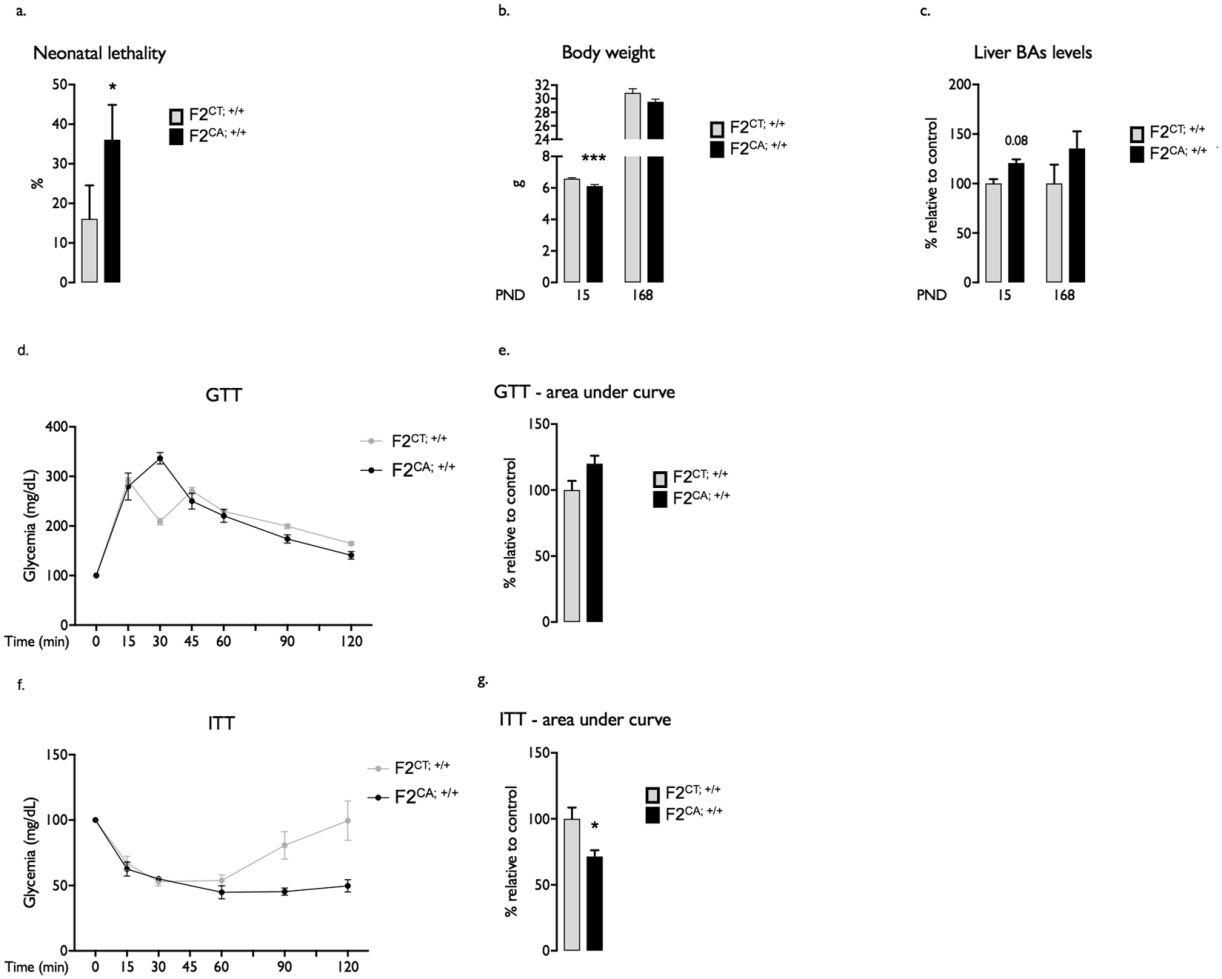
Paternal exposure to CA-diet impacts F2 offspring. (**a**) Percentage of neonatal mortality in F2^CA;+/+^ and F2^CT;+/+^ males. (**b**) Body weight of F2^CA; +/+^ and F2^CT;+/+^ mice at 0, 15,168 postnatal days. (**c**) Hepatic bile acid levels in 15-days old and adult F2^CA;+/+^ and F2^CT;+/+^ mice. (**d**) GTT in adult F2^CA; +/+^ and F2^CT;+/+^ mice. (**e**) Area under the curve for GTT. (**f**) ITT in adult F2^CA; +/+^ and F2^CT;+/+^ mice. (**g**) Area under the curve for ITT. Data are expressed as means +/− SEM. In all panels for each group, *n* = 10–20 males from 3 to 5 independent experiments; *significance; *p* < 0.05.

CA exposure of F0 founders led to postnatal mortality in the corresponding F2 (F2^CA;+/+^) generation similarly to what was observed in the F1^CA;+/+^ offspring (Fig. 3a). At PND15, the surviving F2^CA;+/+^ showed lower body weight (Fig. 3b), associated with a clear but not significant increase in hepatic BA levels compared to the F2^CT;+/+^ males (Fig. 3c). At adulthood, if no difference in glucose tolerance was detected (Fig. 3d,e), F2^CA;+/+^ males showed enhanced sensitivity to exogenous insulin during ITT compared to F2^CT;+/+^ animals (Fig. 3f,g). These results show that paternal CA-exposure resulted in phenotypic abnormalities up to the F2^CA;+/+^ generation.

Breeding of F0^−/−^ males with C57BL/6J females leads to genetic heterogeneity in the following generations. The analysis of these offspring is thus sub-optimal to establish if Tgr5 signaling is involved in the initiation of the multigenerational phenotypes resulting from paternal CA exposure. To encompass this limitation, we used a diet supplemented with oleanolic acid (0.07%-OA), known as a TGR5 agonist^16^. The OA specificity was confirmed using F0^−/−^ males. In F0^OA;+/+^ males, activation of Tgr5 pathways after exposure to OA-diet led to lower sperm count and decreased fertility compared to control males (Supplemental Fig. 2a,b). Similarly to CA-diet, paternal exposure to OA-diet induced perinatal mortality, altered body weight at PND15, and reduced glucose tolerance in adult F1^OA;+/+^ males compared to F1^CT;+/+^ males (Supplemental Fig. 2c–e). Note that OA exposure has no effect on offspring originating from F0^−/−^ male founders (Supplemental Fig. 2). Interestingly, the F2^OA;+/+^ generation showed increased neonatal mortality and lower body weight at PND15 compared to F2^CT;+/+^ pups (Supplemental Fig. 2f,g). Altogether, these last results provide evidences of multi-generational impacts of CA initiated in exposed F0 males by Tgr5 dependent pathways.

### CA-exposure alters DNA methylation in F0^+/+^ sperm cells

To understand the molecular mechanisms underlying the initial steps of this new model of multigenerational impacts of ancestral exposure, we next analyzed the effects of CA-diet on germ cell quality with a particular focus on epigenetic integrity.

During the last step of spermatogenesis, namely spermiogenesis, germ cells undergo a global chromatin remodeling characterized by the replacement of most histones by protamines. In elongated spermatids, histone-protamine transition results in a tight compaction of the genome within the nucleus. Interestingly, electronic microscopy on F0 testes sections (Supplemental 3a) clearly showed altered DNA condensation in spermatids from F0^CA;+/+^ males suggesting defects in chromatin composition following CA treatment. However, no alteration in mRNA accumulation of either transition proteins (*Tnp1* & *Tpn2*) or protamines (*Prm1* & *Prm2*) was observed (Supplemental 3b). In addition, no obvious change in H3 and H4 histones accumulation in mature sperm was detected in F0^CA;+/+^ or F0^CA;−/−^ founders compared respectively to F0^CT;+/+^ or F0^CT;−/−^ male mice (Supplemental 3c). These results suggested that histone-protamine signaling might not be critical to mediate the offspring phenotypes induced by paternal CA exposure.

**Figure 4:**
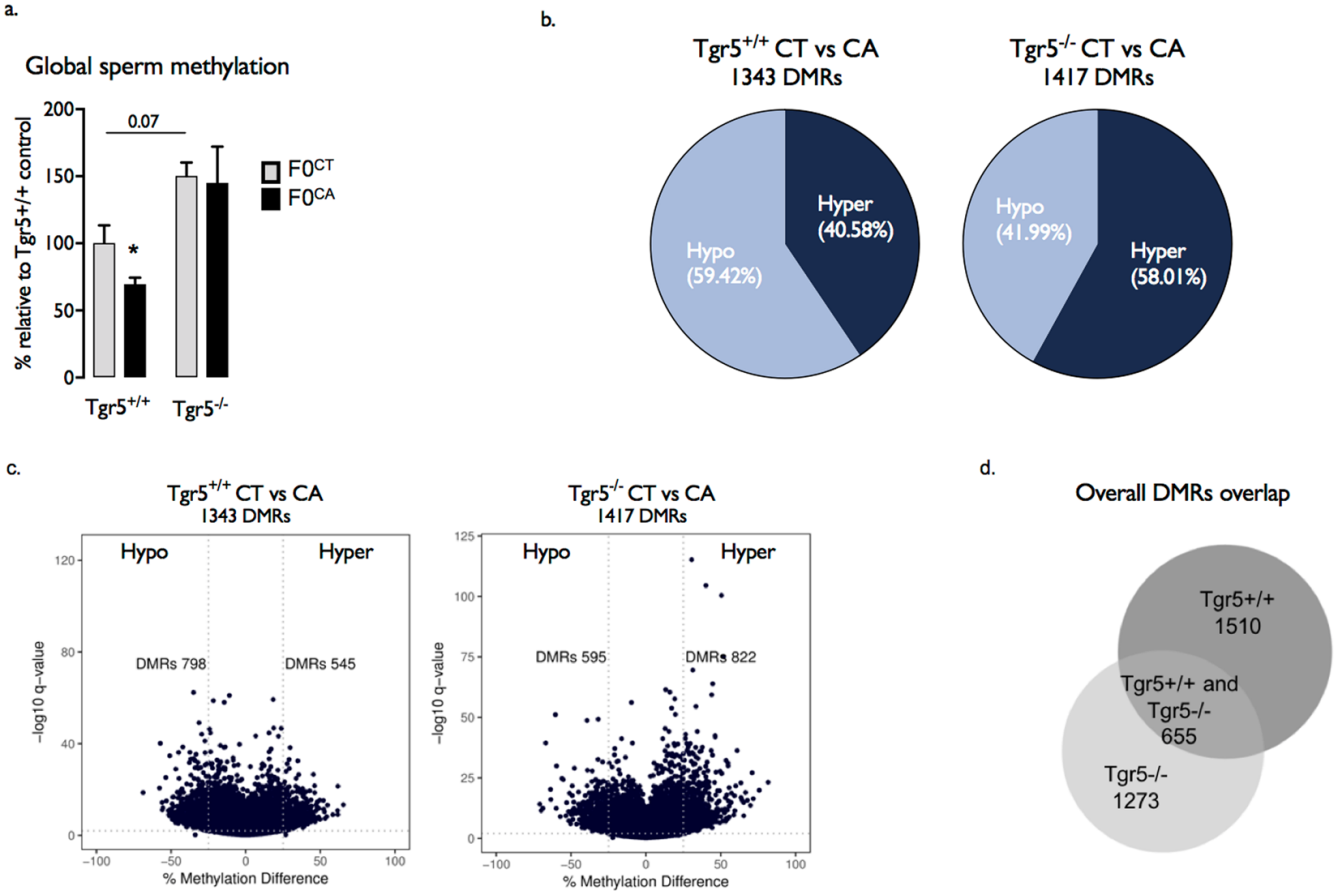
Paternal CA exposure alters global DNA methylation levels in sperm cells. (**a**) Global DNA methylation levels in spermatozoa of F0^CT;+/+^, F0^CA;+/+^; F0^CT;−/−^; and F0^CA;−/−^ mice. (**b**) Pie charts of the relative percentage of hyper- or hypo- DMRs in spermatozoa of F0^CT;+/+^, F0^CA;+/+^; F0^CT;−/−^ and F0^CA;−/−^ mice. (**c**) Volcano plots of comparisons of all DMR assessed in RRBS analysis of spermatozoa from F0^CT;+/+^, F0^CA;+/+^; F0^CT−/−^; and F0^CA;−/−^ mice. (**d**) Representation of specific and common differentially methylated sequences in F0^CT;+/+^, F0^CA;+/+^; F0^CT;−/−^; and F0^CA;−/−^ mice. RRBS was performed on 1 experiment on 3 samples per group. Each sample is a pool of 3 individual mice. For a data are expressed as means +/− SEM. *n* = 8–12 per group from 3 independent experiments. *p < 0.05 vs. control diet group.

Besides histone patterns, sperm DNA methylation is known to play a major role in developmental programing of diseases. Interestingly, mature sperm cells from CA exposed founders (F0^CA;+/+^) showed global DNA hypo-methylation compared to those from F0^CT;+/+^ founders (Fig. 4a). In contrast, no global influence of CA exposure on DNA methylation levels in F0^CA;−/−^ sperm cells was observed (Fig. 4a). The interplay between TGR5 signaling pathways and sperm cell genome methylation was further supported by the increased abundance of 5-methyl-Cytosine (5meC) in F0^CT;−/−^ males compared to F0^CT;+/+^ male founders fed a control diet (Fig. 4a).

Conclusions from global DNA methylation analysis has been confirmed and implemented by a reduced representation bisulfite sequencing (RRBS) approach realized on similar sperm DNA extracts (Fig. 4b,c). DMRs between CA-exposed and control founders were defined as regions containing CpG dinucleotides within a 1000 bp window showing a concordant ≥25% methylation change with a q-value cutoff of 0.01.

In F0^CA;+/+^ sperm cells, 1343 CA-associated DMRs were identified compared to F0^CT;+/+^ group (Fig. 4b,c). The range of differential methylation across these DMRs was 25–68%. Consistent with global hypo-methylation, a majority of 798 hypo-methylated DMRs (59.41% total DMRs) were identified, whereas hyper-methylated DMRs showed 545 occurrences (40.58% total DMRs) (Fig. 4b,c).

Applying the same criteria for calling DMRs, RRBS analysis performed on F0^CA;−/−^ sperm showed 1417 CA-associated DMRs compared to F0^CT;−/−^ sperm cells (Fig. 4b,c) with a range of differential methylation of 25–81%. While F0^CA;+/+^ showed a majority of hypo-methylated DMRs, F0^CA;−/−^ DMRs were mostly hyper-methylated (58.01%) confirming our previous observations on global DNA methylation (Fig. 4b,c).

### Functional analysis of RRBS reveals DMRs associated genes relevant with the multigenerational phenotypes induced by paternal CA exposure

To understand how CA-associated methylation changes might have functional significance in the future embryo, we identified all annotated genes that directly overlapped at least one CpG showing 25% of differential methylation (Supplemental Tables 1–8). Focusing on gene boundaries (intron, exon) and their corresponding promoters; differentially methylated CpGs from F0^CA;+/+^ spermatozoa are contained within a total of 2165 annotated genes. In F0^CA;−/−^ spermatozoa, 1928 differentially methylated genes were identified (Fig. 4d).

Interestingly, the majority of differentially methylated genes determined in F0^CA;+/+^ sperm cells (69.74%) did not overlap with the ones observed in F0^CA;−/−^ sperm cells (Fig. 4d). This set of 1510 annotated genes, specific to exposure of F0^CA;+/+^ males, might be of particular interest to explain the molecular basis of the multigenerational phenotypes induced by paternal exposure to CA-diet.

To gain functional insight, these 1510 associated genes, specifically observed on F0^CA;+/+^ sperm cells were analyzed for Biological process over-representation using PANTHER. Among the 7 categories statistically over-represented after applying the Bonferroni correction for multiple testing, 5 were associated with developmental processes. Of note, “embryonic development” presented the highest enrichment fold (+2.45) (Supplemental Fig. 4).

Additional comparisons for common functional annotations pathways were tested using Enrichr. According to the most updated KEGG database, genes associated with differential CpG methylation showed significant overlap with critical metabolic signaling including “insulin secretion” (P-value = 0.0006) and “glycolysis/gluconeogenesis” (P-value = 0.0280) (Supplemental Fig. 4).

Besides enrichment analysis, genes associated with differential CpG methylation only in F0^CA;+/+^ spermatozoa, were considered for their relevance with abnormalities described in offspring from CA-diet exposed males. This *a priori* read out of the RRBS dataset revealed altered methylation patterns associated with *Fgfr4* and *Klotho*, genes encoding two critical regulators of BA homeostasis^17^.

Our next interest focused on the specific family of imprinted genes. Imprinted genes are defined by allele specific expression that relies on asymmetric 5meC patterns established on imprinted control region (ICR) during gametogenesis. This set of genes is thus of interest in the context of germ cell DNA methylation changes in response to CA exposure. Out of a list of 84 known imprinted genes, 6 were overlapping differentially methylated CpGs in sperm cells from F0^CA;+/+^ males. Those genes include *Peg10*, *Phlda2*, *Plagl1*, or *Kcnq1*. Interestingly, improper dosage of *Peg10* and *Phlda2* are associated with defects in fetal and placental growth as well as sudden perinatal death in mouse. Of similar interest, *Plagl1* or *Kcnq1* dysregulation are responsible for impaired glucose tolerance associated with insulin secretion defects^18,19^.

Altogether, these functional interpretations are consistent with developmental and metabolic abnormalities described in offspring from CA exposed males. This suggests that sperm cells epigenome and more specifically defects in DNA methylation might contribute to the initiation of the phenotypic impacts of CA-diet across multiple generations.

### CA associated DMRs in sperm cells are associated with transcriptional deregulation of functionally related genes in the liver of F1 and F2 offspring

We next asked whether differential methylation in F0 sperm cells might prime the embryo to persistent transcriptomic deregulations underlying phenotypic traits shared across F1 and F2 progenies.

As a representative read out of metabolic defects, total RNA from the liver of both PND15 and/or adult offspring were tested for relative abundance using quantitative PCR. We first tested for transcript deregulation of genes associated with differential CpG methylation in sperm cells of CA treated F0 male founders. As similar deregulations between F0 sperm cells and offspring somatic tissue cannot be strictly expected, we also expended our analysis to a set of genes functionally related to those submitted to DNA methylation change in the CA exposed F0 germline.

**Figure 5:**
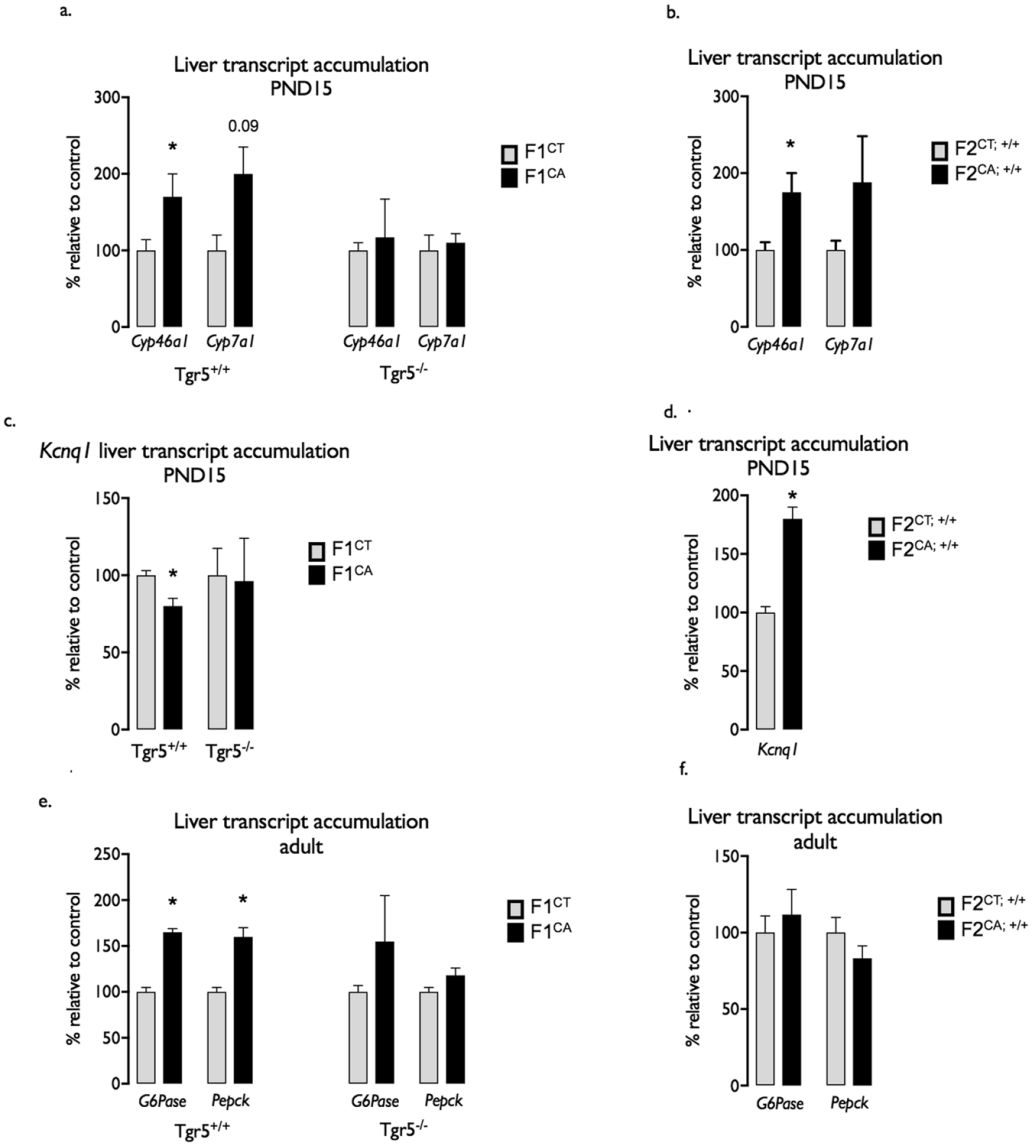
F1^CA;+/+^ males have altered BA metabolism. (**a**) Liver mRNA accumulation of *Cyp46a1* and *Cyp7a1* normalized to *β-actin* mRNA levels in 15-days old F1^CT;+/+^, F1^CT;−/−^, F1^CA;+/+^ and F1^CA;−/−^ mice. (**b**) Liver mRNA accumulation of *Cyp46a1* and *Cyp7a1* normalized to *β-actin* mRNA levels in 15-days old F2^CT;+/+^ and F2^CA;+/+^ mice. (**c**) Liver mRNA accumulation of *Kcnq1* normalized to *β-actin* mRNA levels in 15-days old F1^CT;+/+^, F1^CT;−/−^, F1^CA;+/+^ and F1^CA;−/−^ mice. (**d**) Liver mRNA accumulation of *Kcnq1* normalized to *β-actin* mRNA levels in 15-days old F2^CT;+/+^ and F2^CA;+/+^ mice. (**e**) Liver mRNA accumulation of *G6Pase* and *Pepck* normalized to *β-actin* mRNA levels in adult F1^CT;+/+^, F1^CT;−/−^, F1^CA;+/+^ and F1^CA;−/−^mice. (**f**) Liver mRNA accumulation of *G6Pase* and *Pepck* normalized to *β-actin* mRNA levels in 15-days old F2^CT;+/+^ and F2^CA;+/+^ mice. Data are expressed as means +/− SEM. *n* = 8–12 per group from 3 independent experiments. *p < 0.05 vs. control diet group.

At PND15, although hepatic transcript levels of *Fgfr4* and *Klotho* were not altered (Supplemental Fig. 5), accumulation of *Cyp46a1* and *Cyp7a1* were increased in F1^CA;+/+^; whereas no effect was observed in F1^CA;−/−^ males compared to F1^CT;−/−^ males (Fig. 5a). Same deregulation was observed in the liver of 15 days old F2^CA;+/+^ progeny originating from CA exposed grandfathers (F0^CA;+/+^) comared to F2^CT;+/+^ males (Fig. 5b). *Cyp46a1* and *Cyp7a1* are encoding for enzymes directly involved in primary BAs synthesis. The functional significance of their transcript deregulation was supported by increased hepatic BAs levels measured in 2 weeks old F1^CA;+/+^ and F2^CA;+/+^ progenies from CA exposed F0 compared to control males (Fig. 2).

Always at PND15, *Kcnq1* an imprinted gene associated with DMRs in F0 sperm cells, showed transcript deregulations in the liver of F1^CA;+/+^ and F2^CA;+/+^ offspring when compared to their respective controls (Fig. 5c,d). In the F1 generation, the decrease in *Kcnq1* was dependent on paternal Tgr5 signaling as F1^CA;−/−^ male transcript levels were not affected compared to F1^CT;−/−^ (Fig. 5c).

In line with defects in glucose homeostasis, adult F1^CA;+/+^ males originated from CA exposed F0^CA;+/+^ founders showed consistent deregulation of *G6Pase* and *Pepck* mRNA accumulations compared with controls (Fig. 5e). No alteration of *G6Pase* and *Pepck* expression was observed in F1^CA;−/−^ males compared to F1^CT;−/−^ males (Fig. 5e). If insulin response was altered in F2^CA;+/+^ males, those individuals did not show any difference in hepatic mRNA accumulations of *G6Pase* and *Pepck* when compared to their respective control group (Fig. 5f). These data suggest that molecular mechanisms specific to each generation underlie common metabolic traits resulting from paternal CA-diet exposure.

### *Tgr5* dependent regulation of *Dnmt3b* within the germ cell lineage is a strong candidate to prime the phenotypic impacts of paternal CA exposure across multiple generations

**Figure 6:**
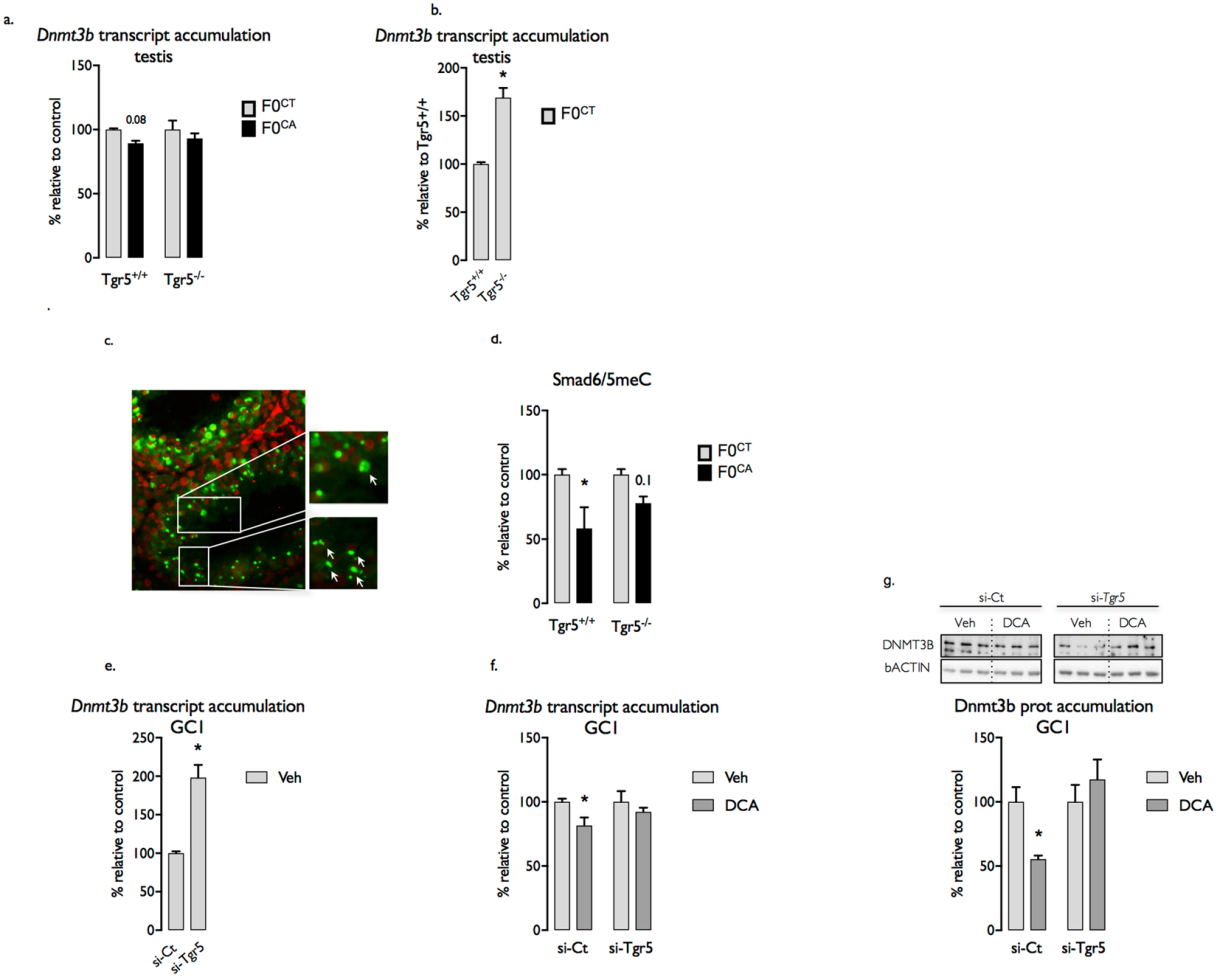
Adult CA-exposure alters male germ cells. (**a**) Testicular mRNA accumulation of *Dnmt3b* normalized to *β-actin* mRNA levels in adult F0^CT;+/+^ and F0^CT;−/−^ male founders. (**b**) Testicular mRNA accumulation of *Dnmt3b* normalized to *β-actin* mRNA levels in adult F0^CT;+/+^; F0^CT;−/−^; F0^CA;+/+^ and F0^CA;−/−^ male founders. (**c**) Immunohistochemistry of Smad6 and 5meC in testis of F0^CT;+/+^; F0^CT;−/−^; F0^CA;+/+^ and F0^CA;−/−^ male founders. (**d**) Quantification of the relative percentage of seminiferous tubules with germ cells co-stained for Smad6 and 5meC in testis of F0^CT;+/+^; F0^CT;−/−^; F0^CA;+/+^ and F0^CA;−/−^ male founders. (**e**) mRNA expression of *Dnmt3b* normalized to *β-actin* levels in GC1spg germ cell lines transfected with siGfp or siTgr5. (**f**) mRNA expression of *Dnmt3b* normalized to *β-actin* levels in GC1spg germ cell lines transfected with siGfp or siTgr5 and exposed to vehicle or DCA during 24 hours. (**g**) DNMT3B protein accumulation normalized to *ACTIN* levels in GC1spg germ cell lines transfected with siGfp or siTgr5 and exposed to vehicle or DCA during 24 hours. Data are expressed as means +/− SEM. *n* = 8–15 per group from 3 independent experiments. In a, b, c and d: *p < 0.05 vs. control diet group. In e, f and g: *Difference from the siGfp vehicle group; ^#^difference from the siGfp DCA group. siGfp vehicle-treated cells were fixed at 100% for each siRNA condition.

To understand how Tgr5 signaling pathways affect germ cell DNA methylation, we analyzed testicular abundance of transcripts encoding for DNA-methyltransferases (Dnmts) (Fig. 6a and Supplemental 6). No impact of CA-diet was observed for *Dnmt1*, *Dnmt3a* and *Dnmt3L* (Supplemental 6), whereas a decrease of *Dnmt3b* mRNA accumulation was detected in testis of F0^CA;+/+^ compared to F0^CT;+/+^ founders (Fig. 6a). Of note, *Dnmt3b* mRNA accumulation remained unaffected in testis of F0^CA;−/−^ males compared to their respective controls (Fig. 6b). Interestingly, F0^CT;−/−^ mice showed an increased *Dnmt3b* mRNA accumulation compared to F0^CT;+/+^ founders (Fig. 6b). These results are consistent with previous quantification of global sperm DNA methylation, as well as the RRBS dataset obtained from F0 sperm.

*Dnmt3b* is expressed in different cell types within the testis such as Leydig, Sertoli and germ cells^7,20,21^. To define if the observed deregulation of testicular *Dnmt3b* mRNA accumulation could be correlated with the impacts of CA exposure on DNA methylation levels within germ cell lineage, we performed immunohistochemistry for co-staining of Smad6, a marker of post-meiotic germ cells, and 5-methylcytosine (5meC). Consistently with the observed sperm cell DNA hypo-methylation, F0^CA;+/+^ males showed a lower proportion of seminiferous tubules containing post-meiotic germ cells co-stained for Smad6 and 5meC compared to F0^CT;+/+^ males (Fig. 6c,d). This effect was not observed in the testis of F0^CA;−/−^ when compared to F0^CT;−/−^ group (Fig. 6d).

The use of the spermatogonial cell line GC1-spg allowed demonstrating that Tgr5 signaling pathways control *Dnmt3b* expression within germ cell lineage. Indeed, GC1spg cells transfected with a siRNA directed against Tgr5 showed higher mRNA accumulation of *Dnmt3b* compared to cells transfected with a control siRNA (Fig. 6e). Moreover, treatment of GC1-spg cells with deoxycholic acid (DCA) induced a significant decrease of *Dnmt3b* while transcript accumulation remained stable in cells transfected with a siRNA directed against Tgr5 (Fig. 6f). Those significant transcriptomic changes have been confirmed at the protein level (Fig. 6g). Altogether these results suggest that Tgr5 dependent regulation of Dnmt3b within germ cells can impact sperm DNA methylation and prime the phenotypic impacts of paternal BA exposure across multiple generations.

## Discussion

A number of experimental animal models have shown that paternal exposure to metabolic stress can have a significant influence on the susceptibility of future generations to diseases.

**Figure 7:**
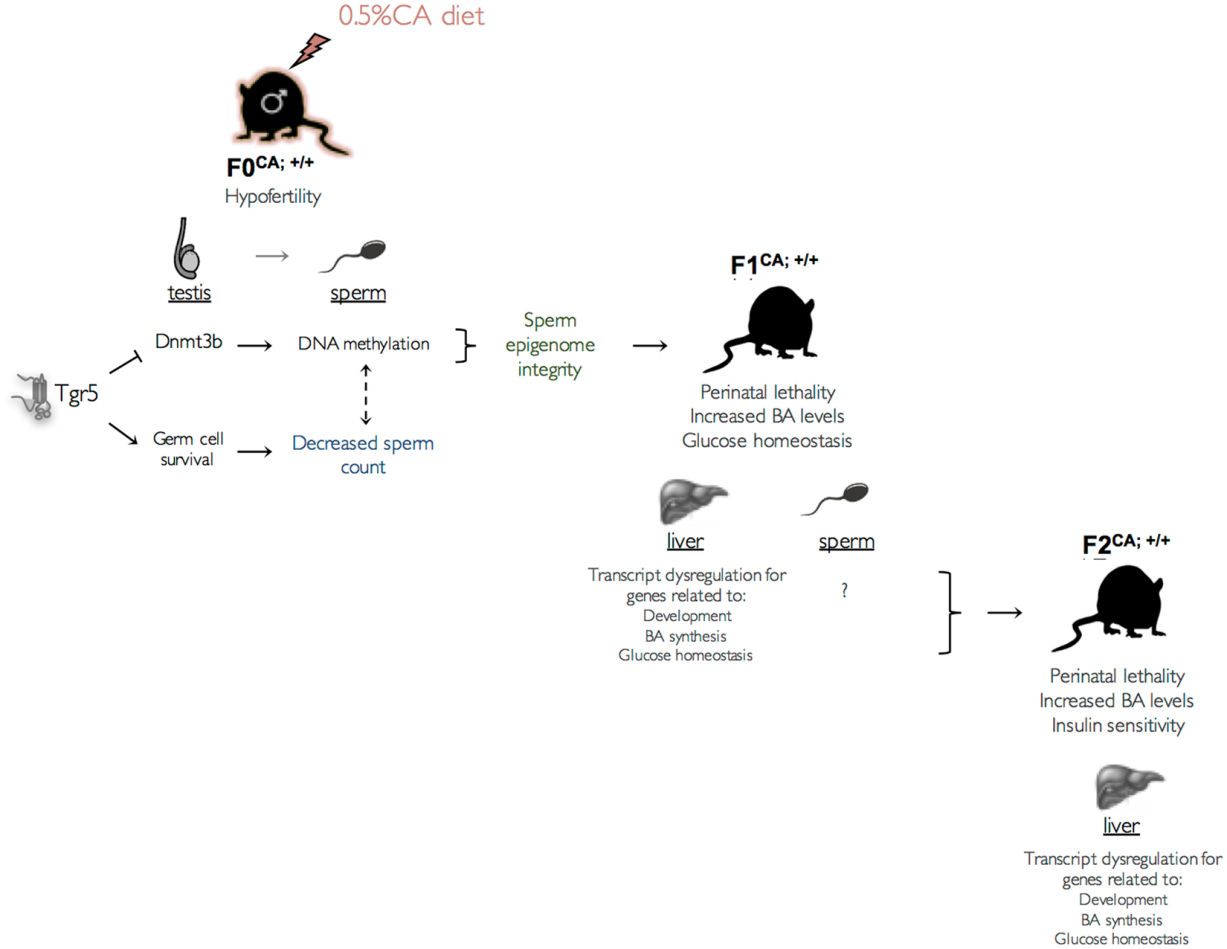
Proposed scheme of the transgenerationnal impacts of paternal CA-diet exposure.

In the present study, we demonstrate that pathological concentrations of BA are responsible for multigenerational phenotypes (Fig. 7). Two generations of progenies from males exposed to CA-diet show developmental and metabolic abnormalities. These defects are associated with differential DNA methylation in the mature sperm of CA-exposed males. The main contribution of paternal Tgr5 signaling is supported by the fact that progeny originated from F0^CA;−/−^ founders are protected from the adverse effects of paternal exposure to CA-diet. Further explorations suggest that Tgr5 dependent regulation of *Dnmt3b* expression within germ cells may constitute the primary molecular event linking paternal BA exposure to the epigenetic programming of diseases across multiple generations.

Our previous research identifies BAs as reprotoxic molecules affecting sperm number^14^. Testicular defects associated with the phenotype included a loss of cell junctions within the seminiferous epithelium as well as an increased cell death affecting advance stages of spermatogenesis. Those observations were dependent upon the activation of the membrane BA receptor Tgr5 within the germ cell lineage. Indeed, mice presenting a global deletion of Tgr5^−/−^ were protected against fertility damages associated with BA exposure. In this present study, we show that the deletion of Tgr5 also protect progenies to negative phenotypic outcomes induced by paternal CA exposure.

These results were unexpected, as it has been demonstrated that upon CA-supplemented diet, the absence of Tgr5 worsens liver injury creating systemic conditions that are themselves responsible for reproductive abnormalities. Interestingly, hepatic parameters known to impact male fertility such as the Insulin-growth-factor-1 (Igf1), and enzymes responsible for the catabolism of testosterone in hepatocytes, were similarly affected in F0^CA;+/+^ and F0^CA;−/−^ males in response to CA-diet exposure^14^. This is the demonstration that systemic changes associated with CA treatment in both genetic backgrounds can’t explain the reproductive defects, which remained unique to wild-type mice.

We then raised the hypothesis that BA, without systemic intermediates, may act directly within the testis *via* Tgr5 dependent signaling pathways. In this study, the use of the spermatogonial cell line GC1-spg confirmed the activity of Tgr5 for the regulation of *Dnmt3b* expression within the germ cell lineage.

In conclusion and despite the limitations of systemic changes associated with non-tissue-specific gene deletion, Tgr5 knock-out mice appear to be a reliable model to define the impacts of CA-diet on testis physiology. However, genetic heterogeneity of progenies originating from the mating of F0^−/−^ males with wild-type females raised the limitations of our model to explore generational phenotype resulting from paternal BA exposure (in the following generations). We then used alternative strategy allowing genetic consistency while focusing on Tgr5 specificity. Feeding F0^+/+^ and F0^−/−^ males with oleanolic acid, a specific Tgr5 agonist, confirms the critical role of Tgr5 signaling to program phenotypic outcomes up to the second generation.

Adult exposure to CA-diet is known to reduce sperm count and decrease fertility rates. Beside the quantitative impact of BAs on reproductive function, the present study demonstrate that pathological concentrations of BAs are responsible for germ cell epigenetic abnormalities and disease programming on two generations of offspring. The last few years have seen increasing interest in investigating the epigenetic dimension of male fertility defects. Recently, human studies along with experimental model in rodents, describe a strong association between reproductive disorders and sperm epimutations. A good illustration come from recent work showing that sperm collected for the purpose of Assisted Reproductive Technologies (ART) present abnormal chromatin composition as well as altered DNA methylation patterns^22^. In mouse, exposure to endocrine disruptors such as vinclozoline, a fungicide known for its repro-toxic properties, increases spermatogenic cell apoptosis rate and alters sperm DNA methylation^23^. Interestingly, ART or paternal exposures to vinclozolin have both been associated with offspring phenotypes across multiple generations^22^. In this context, the impacts of supra-physiological BA concentrations on sperm epigenome define an original model of paternal inheritance where metabolic compounds can affect offspring.

Three major classes of epigenetic information remain the likeliest candidates to carry paternal information to offspring. Those include cytosine methylation, chromatin structure, and non-coding RNA.

In the present study, electronic microscopy realized on testes sections clearly showed altered DNA condensation of spermatids in response to CA-diet exposure. Several reports link this feature to an alteration of the histone-protamine transition during spermiogenesis that would ultimately lead to abnormal retention of nucleosomes in mature sperm^23,24^. Even if our model of CA exposure is clearly associated to the observation of uncondensed spermatid nuclei, we were not able to detect substantial chromatin change in mature spermatozoa. The elimination of the most affected spermatids prior their release into the epididymis would explain this interesting discrepancy. Supporting this hypothesis, we previously demonstrate that CA exposure is responsible to spermatid cell death. Further investigations using sensitive approaches must be considered to highlight potential subtle changes in chromatin geography or composition in spermatozoa that survived germ cell selection.

The major epigenetic change detected in sperm in response to CA exposure concern DNA methylation levels. A first approach allowed us to identify a 30% decrease in global 5meC on sperm from F0^CA;+/+^ compared to controls. Interestingly several studies report that global hypo-methylation are correlated with poor pregnancy outcomes in IVF patients^25^. Consistently, reduced sperm DNA methylation is associated with decreased fertility in our model of BA exposure.

Changes in sperm DNA methylation are also known to affect the proper initiation of the transcriptional program of the zygote. As a consequence, paternal DMRs have the potential to interfere with the early processes of cell differentiation and ultimately with the functional determination of embryonic tissues. Sperm DMRs are thus strong candidates to initiate a cascade of molecular alterations increasing susceptibility to diseases in later life.

In our model, changes in sperm DNA methylation induced by CA exposure are associated with high rates of neonatal lethality in the corresponding F1 and F2 progenies. Macroscopic observations of dead newborns reveal a broad range of phenotypic abnormalities. Further experimentations would be useful to characterize the nature of these developmental disorders to understand their origin. Later in life, surviving offspring develop metabolic syndromes associated with increased BA concentrations as well as defect in glucose/insulin homeostasis. Altogether these data demonstrate that differential methylations in the sperm cells from CA exposed males are associated with phenotypic outcomes in two generation of progenies.

Understanding the mechanistic relationship between sperm DMRs and multigenerational phenotypes constitute a real challenge. Many examples in the field demonstrate that differential methylations in sperm might not be strictly maintained in F1 tissues due to the dramatic epigenetic programming required for cell fate determination during development. However, sperm DMRs are expected to influence the expression of gene networks functionally related to those submitted to sperm DNA methylation changes.

Results obtained in the context of CA exposure are consistent with this model. We identified 2160 genes associated with differential CpG methylations in the sperm of F0^CA;+/+^. Among them, 1510 were specific to F0^CA;+/+^ males. Even if the majority of them did not show differences in expression in the liver of the corresponding F1 and F2, enrichment analysis show that sperm differentially methylated genes are functionally relevant with transcriptional changes associated with offspring phenotypes.

In this manuscript we demonstrate that paternal CA exposure can reproduce similar phenotypic traits up to the second generation through the male germline. This new example of transgenerational inheritance prompts us to understand how F1 spermatozoa could in turn carry the memory of ancestral CA exposure. In this study, we focused our interest on sperm DNA methylation. Global 5meC levels were clearly but not statistically decreased in sperm from F1^CA;+/+^ compared to F1^CT;+/+^ males (data not shown). Further analysis would be required to decipher if F1 sperm present similar epigenetic changes than the one observed in F0 germ cells directly exposed to CA diet. Few examples in the literature show that differential methylation can be maintained in the male germline across several generations by escaping the primordial germ cells reprogramming. Another hypothesis would be that the phenotypic traits observed in the first generation of offspring are able to reproduce similar epigenetic alterations on their own germ cells.

The use of Tgr5 knock out mice as well as spermatogonial germ cell *in vitro*, shown that the activation of Tgr5 signaling by BA reduces *Dnmt3b* expression. Previous reports have shown similar deregulations of *Dnmt3b* expression in response to various reprotoxic molecules. In male rat, neonatal exposure to xeno-estrogens leads to decrease Dnmt3b levels in the germ cells leading ultimately to reduce DNA methylation^23^. In our model, Tgr5 dependent regulation of *Dnmt3b* may constitute the primary molecular event linking paternal CA exposure, sperm hypo-methylation and developmental programming of diseases across multiple generations.

Our study provides fundamental findings that may contribute to a better understanding of how paternal exposure to stressors can shape offspring physiology through several generations.

Altered BA homeostasis is the direct consequence of prevalent metabolic diseases affecting our society worldwide. Along with liver failure, intestinal inflammation and obesity are all known to impact BA levels and biochemical composition.

Beside their repro-toxic property, this study identified BAs as critical mediators of transgenerational metabolic diseases impregnation. Our conclusions will highlight important opportunities for prevention and therapeutic intervention in order to slow the growing epidemic of decreased sperm quality and its consequences on metabolic disease programing across generations.

## Methods

### Animals

Nine-week-old *Tgr5*^+/+^*(F0*^+/+^) and *Tgr5*^−/−^ *(F0*^−/−^) mice used have been described elsewhere^14^. The mice used in this study were maintained as a C57BL/6J background and housed in temperature-controlled rooms with 12 h light/dark cycles. Mice had *ad libitum* access to food and water. Nine-week-old mice F0 founders were exposed to the D04 diet (Control) or the D04 diet, supplemented with 0.5% cholic acid (CA-diet) (SAFE, Augy, France) for 4 months. The experimental procedure to study the multi-generational transmission from F0 to F2 is reported in Fig. 1.

This study was conducted in accordance with standards approved by the local Animal Care Committee (Comté d’Ethique pour l’Expérimentation Animale Auvergne C2E2A; CE07-12 & 08-12).

### Electron microscopy

Electron microscopy was performed as previously described^26^. Briefly, samples were set in 2% glutaraldehyde and 0.5% paraformaldehyde in cacodylate buffer at 4 °C for 24 h. Set testes were subsequently postfixed for 1.5 h in buffered osmium tetraoxide at 4 °C and embedded in Epon Araldite (Delta Microscopies, Ayguesvives, France). Ultrathin sections were stained with uranyl acetate and examined with a Hitachi H-7650 transmission electron microscope (Hitachi Elexience, Verrières-le-Buisson, France).

### Cell Culture

GC1‐spg cells were used as previously described^14^. GC1-spg cells were transfected with siRNA directed against *Tgr5* or control siRNA. 48 hours after the transfection, cells were treated for 24 hours with vehicle or DCA (10 µM).

### Glucose and insulin measurements

Colorimetric assays were performed as recommended by the manufacturer (Glucose RTU, 61269, Biomerieux SA, France). Insulin measurements were performed using an EIA kit (A05105, Bertin Pharma, France).

### Glucose tolerance test

Mice were fasted overnight. They were then challenged with glucose (2 g/kg). Blood glucose concentrations were measured at regular time points.

### Insulin tolerance test

Mice were fasted for 4 h and then challenged with insulin (1mU/g) by IP. Blood glucose concentrations were measured at regular time points, and blood samples were collected to determine insulin levels.

### Real-time RT-PCR

RNA from testis samples was isolated using Nucleospin RNA L (Macherey-Nagel, Hoerdt, France). cDNA was synthesized from total RNA with the MMLV reverse transcriptase and random hexamer primers (Promega, Charbonnière-les-Bains, France). The real-time PCR measurement of individual cDNAs was performed using SYBR green dye (Master mix Plus for SYBR Assay, Eurogentec, Angers, France) to measure duplex DNA formation with the Eppendorf Realplex system. The sequences of primers are the following: Primers were used previously in the following articles^14,27,28^ or in Supplemental Table S9. Standard curves were generated with pools of testis cDNA from animals with different genotypes and/or treatments. The results were analyzed using the ΔΔct method.

### Spermatozoa purification

The cauda sperm isolation has been designed according to relevant protocols described in the literature. The cauda epididymides were gently squeezed to allow the caudal fluid to ooze out. After 15 minutes incubation at 37 °C, swimming sperm containing in the media were transferred to a new tube. Sperm were collected by centrifugation at 2000 × g for 2 minutes, followed by two 1X PBS wash before freezing down. Sperm sample purity was confirmed by microscopic examination. The purity of the sperm was evaluated at the sacrifice when the sperm cells were collected. The overall percentage of purity was around 95% to 99%.

### DNA methylation analysis

DNA was extracted from sperm cells, and global methylation levels were then quantified using an ELISA kit (P-1014B-48, Euromedex).

### Reduced representation bisulfite sequencing (RRBS)

RRBS was performed by Diagenode. Sequencing on an Illumina HiSeq3000. Quality control of sequencing reads was performed using FastQC. Adapter removal was performed using Trim Galore! version 0.4.1. Reads were then aligned to the reference genome using bismark v0.16 software, followed by methylation calling using the corresponding bismark function. The comparisons between the RRBS data sets were carried out using methylKi, with the mm10 refGene and CpG island annotation from UCSC. Differentially methylated CpGs, and differentially methylated regions (DMRs) were identified (the latter with a window size of 1000 bp, which had been found to include most DMRs). Both differentially methylated CpGs and differentially methylated regions (width 1000 nts) were identified for the data set, with a percent methylation difference cutoff of 25% and a *q*-value of 0.01. The percentages of hypo- and hypermethylated CpGs were plotted on a bar chart per chromosome. Additionally, the differentially methylated CpGs were annotated with different genomic regions, along with CpG island and shore coordinates and their distribution.

### Immunohistochemistry

5 μm paraffin sections of testes fixed with PFA4% solution were performed and mounted on positively charged glass slides (Superfrost plus), deparaffinized, rehydrated, treated for 20 min at 93°–98 °C in 0.01 M citric buffer (pH 6), rinsed in osmosed water (2 × 5 min), and washed (2 × 5 min) in Tris-buffered saline (TBS). Immunohistochemical studies were conducted according to the manufacturer’s recommendations, as described earlier^29^. Slides were then counterstained with Hoechst medium (1 mg/mL). The antibodies used were Smad6 (Santa Cruz; sc 6034) and 5meC (Abcam; ab10805).

### Western Blot

Proteins were extracted from tissues using lysis buffer (0.4 M NaCl, 20 mM HEPES, 1.5 mM MgCl2, 0.2 mM EDTA, 0.1% NP-40, 1 × protease inhibitors [Roche Diagnostics]). Antibodies were used in TBS, 0.1% Tween, and 10% milk. The antibodies used are H3 (Santa Cruz; sc 8654) and H4 (Cell Signaling; 2935 S) and DNMT3b (Abcam; ab16049).

### Statistics

For statistical analysis, student t-test or ANOVA analysis were performed using the statistical software package SigmaStat 3.0. Data are mean ± SEM. *p* value less than 0.05 was considered significant.

## Electronic supplementary material

supplemental information

RRBS dataset

supplementary table 9
