# Supplementary material for: Multigenerational impacts of bile exposure are mediated by TGR5 signaling pathways
Source: Sci Rep. 2018 Nov 15;8:16875. doi: 10.1038/s41598-018-34863-0 (PMC6237852; doi:10.1038/s41598-018-34863-0)
Supplement: Supplementary file 1 — supplemental information [file 41598_2018_34863_MOESM1_ESM.pdf]

Multigenerational impacts of bile exposure are mediate by TGR5 signaling pathways.

Marine Baptissart[1], Lauriane Sèdes[1], Hélène Holota[1], Laura Thirouard[1], Emmanuelle Martinot[1], Angélique de Haze[1], Betty Rouaisnel[1], Françoise Caira[1], Claude Beaudoin[1] and David H. Volle[1*]

[1]INSERM U1103, Université Clermont Auvergne, CNRS UMR-6293, GReD, F-63000 Clermont–Ferrand, France

Correspondance. D.H. VOLLE. INSERM U1103, CNRS UMR-6293, UCA, GReD, 28 place Henri Dunant, 63001 Clermont-Ferrand, France. Phone: +33-4-73407415; david.volle@inserm.fr.

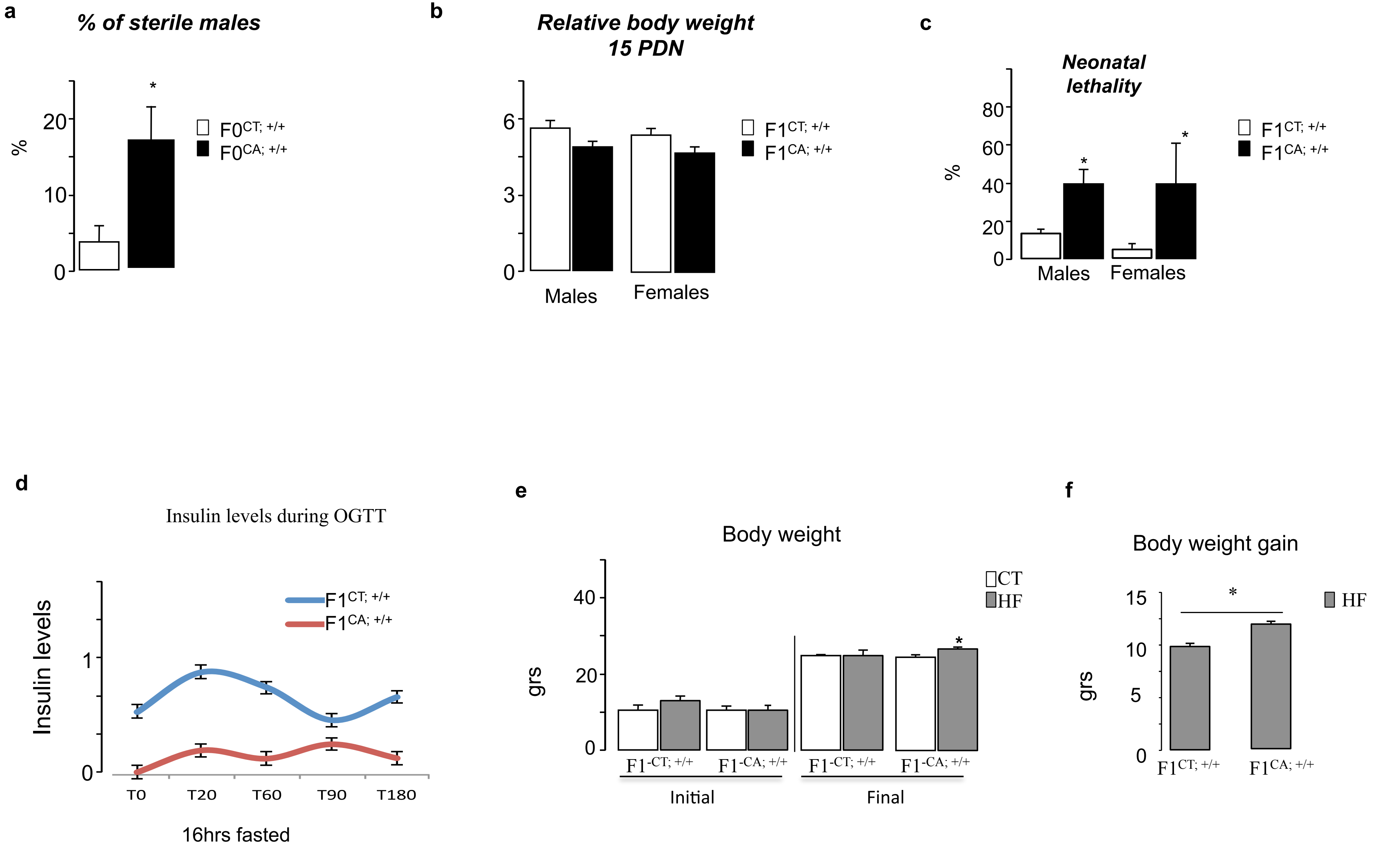

a
% of sterile males
%
F0CT; +/+
F0CA; +/+
b
Relative body weight
15 PDN
Males
Females
F1CT; +/+
F1CA; +/+
c
Neonatal lethality
%
Males
Females
F1CT; +/+
F1CA; +/+
d
Insulin levels during OGTT
Insulin levels
F1CT; +/+
F1CA; +/+
T0
T20
T60
T90
T180
16hrs fasted
e
Body weight
grs
CT
HF
F1-CT; +/+
F1-CA; +/+
Initial
Final
f
Body weight gain
grs
HF
F1CT; +/+
F1CA; +/+

Supplemental-1. Paternal exposure to CA-diet impacts offspring. a/ Fertility of males founders exposed to CT or CA diet. **b**/ Body weight of males and females $F1^{CT;\ +/+}$ and $F1^{CA;\ +/+}$ mice at 15-day-old; **c/** Percentage of neonatal mortality of F1 males and females originating from $F0^{+/+}$ males fed 4 months with CT or CA diet. **d/** Insulin levels during OGTT in $F1^{CT;\ +/+}$ and $F1^{CA;\ +/+}$ mice at adulthood originating respectively from fathers (F0) fed 4 months with Control or 0.5%-CA diet. **e/** Analysis of body weight in $F1^{CT;\ +/+}$ and $F1^{CA;\ +/+}$ males fed 5 weeks with Control or high-fat diet. **f/** Weight gain of $F1^{CT;\ +/+}$ and $F1^{CA\ ;\ +/+}$ males fed 5 weeks with Control or high-fat diet.

Data are expressed as the means +/-SEM. $n = 6–12$ per group from 2 to 4 independent experiments. Statistical analysis: * $p < 0.05$ vs. control diet group.

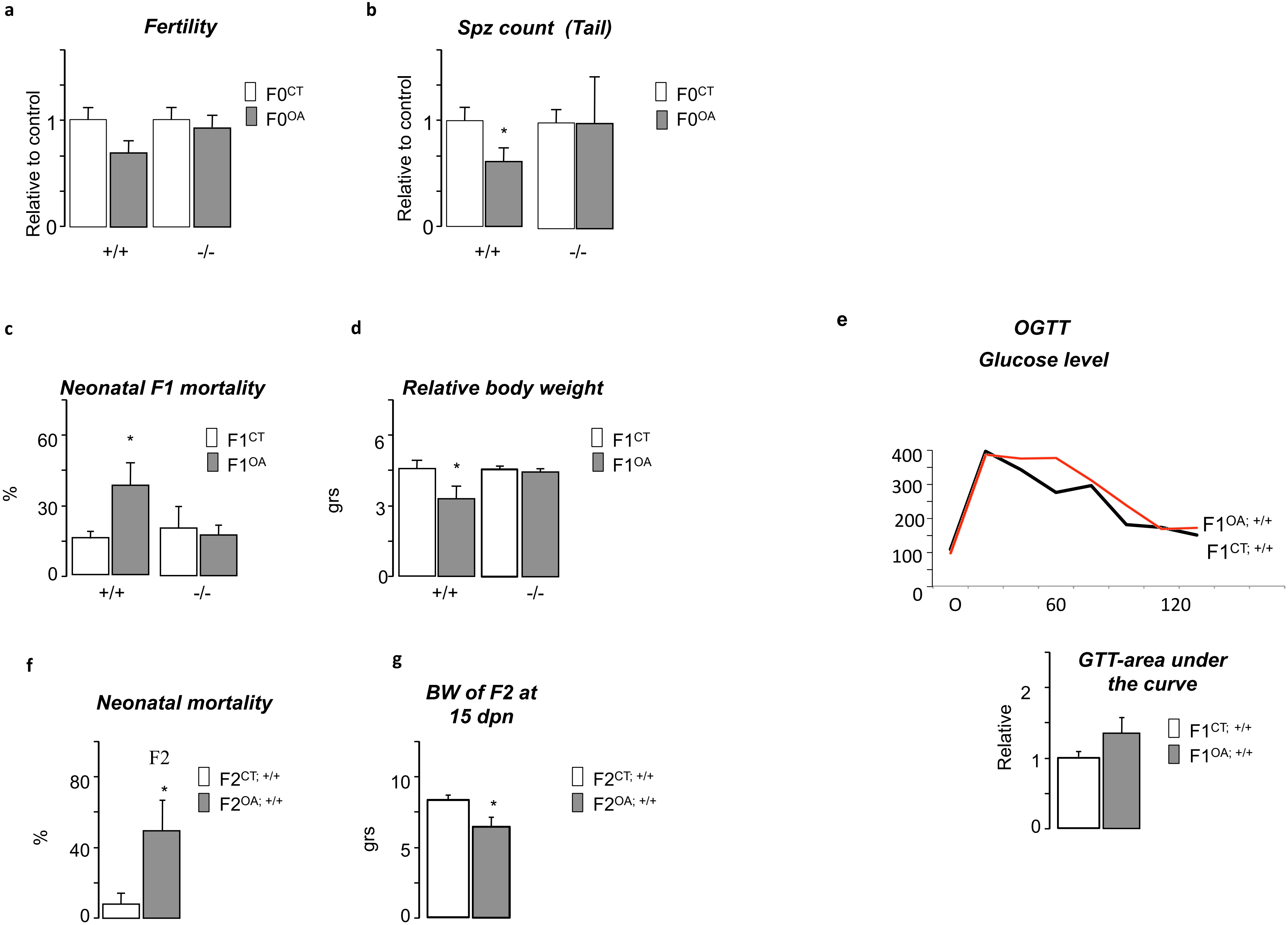
a
Fertility
Relative to control
0
1
F0CT
F0OA
+/+
-/-
b
Spz count (Tail)
Relative to control
0
1
*
F0CT
F0OA
+/+
-/-
c
Neonatal F1 mortality
%
0
30
60
*
F1CT
F1OA
+/+
-/-
d
Relative body weight
grs
0
3
6
*
F1CT
F1OA
+/+
-/-
e
OGTT
Glucose level
0
100
200
300
400
O
60
120
F1OA; +/+
F1CT; +/+
GTT-area under the curve
Relative
0
1
2
F1CT; +/+
F1OA; +/+
f
Neonatal mortality
%
0
40
80
F2
*
F2CT; +/+
F2OA; +/+
g
BW of F2 at 15 dpn
grs
0
5
10
*
F2CT; +/+
F2OA; +/+

Supplemental 2. Impact of paternal exposure to OA diet on F1 and F2 offspring.

a/ Each male was bred with two C57BL/6J females, and the percentage of sterile males was monitored for males fed Control diet or OA diet for 4 months. Control diet group was arbitrarily set at 1 as reference.

b/ Relative number of spermatozoa counts in the epididymis head in F0 males fed Control diet or OA diet for 4 months. Control diet group was arbitrarily set at 1 as reference.

c/ Percentage of neonatal mortality in litters obtained from fathers fed 4 months with Control or OA diet.

d / Body weight of 15-day-old $F1^{CT;+/+}$ and $F1^{OA;\ +/+}$ mice originating respectively from fathers (F0) fed 4 months with Control or OA-diet.

e/ OGTT in adult $F1^{CT;+/+}$ and $F1^{OA;\ +/+}$ mice originating respectively from fathers (F0) fed 4 months with Control or OA-diet.

f/ Percentage neonatal mortality in F2 litters obtained from $F1^{CT;\ ++}$ and $F1^{OA;\ +/+}$.

g/ Body weight in 15-day-oldof $F2^{CT;\ +/+}$ and $F2^{OA;\ +/+}$ mice originating respectively from fathers (F0) fed 4 months with Control or OA-diet.

In all panels data are expressed as means +/-SEM. $n = 6\text{-}15$ per group from 3 independent experiments. Statistical analysis: * $p < 0.05$ vs. control diet group.

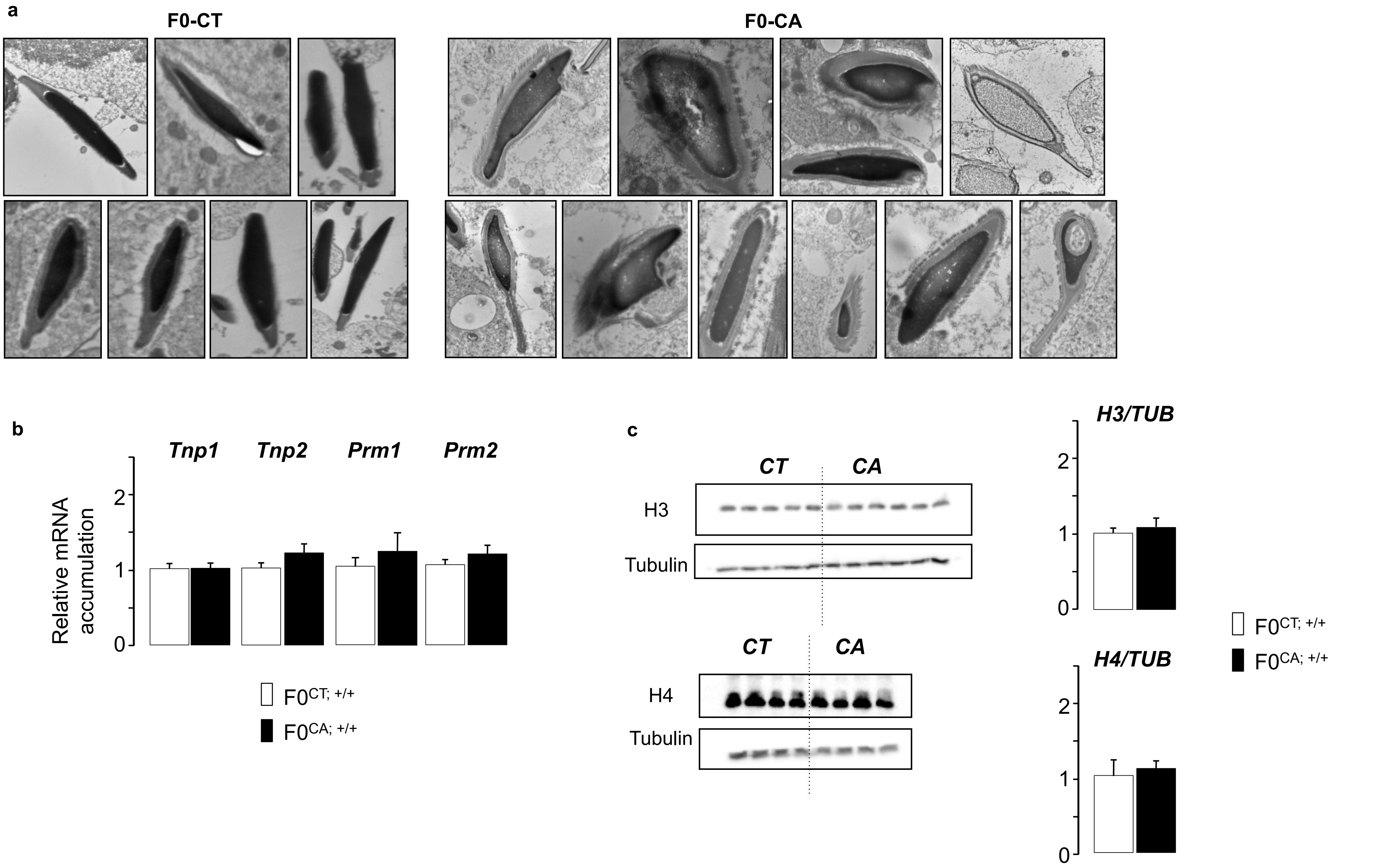
a
F0-CT
F0-CA
b
Tnp1
Tnp2
Prm1
Prm2
Relative mRNA accumulation
0
1
2
F0CT; +/+
F0CA; +/+
c
CT
CA
H3
Tubulin
H4
H3/TUB
H4/TUB

Supplemental 3. Adult CA-exposure alters male germ cells. **a/** Representative electron micrographs of testicular spermatozoa of $F0^{CA;\ +/+}$ and $F0^{CT;\ +/+}$ males. **b/** Testicular mRNA accumulation of *Tnp1*, *Tnp2, Prm1* and *Prm2 in* $F0^{+/+}$ *males fed* Control or 0.5%-CA diet. **b/** Immunoblotting of histone H4, histone H3 and TUBULIN performed on testicular protein extracts of $F0^{CA;\ +/+}$, $F0^{CT;\ +/+}$, $F0^{CT;\ -/-}$, $F0^{CA;\ -/-}$ mice. **c/** Quantification of H3 and H4 compared to TUBULIN. Control-diet-treated mice were arbitrarily set at 1.

In all panels data are expressed as means +/-SEM. $n = 6\text{-}15$ per group from 3 independent experiments. Statistical analysis: * $p < 0.05$ vs. control diet group.

A. PANTHER GO-Slim Biological Process	Number of BA associated DMGs	Over/under representation	Fold Enrichment	P-Value (Bonferroni correction)	Client Text Box Input (expected)
embryo development (GO:0009790)	22	+	2,45	3,82E-02	8,98
synaptic transmission (GO:0007268)	34	+	2,07	2,16E-02	16,45
mesoderm development (GO:0007498)	50	+	1,87	7,87E-03	26,81
nervous system development (GO:0007399)	67	+	1,71	6,22E-03	39,24
system development (GO:0048731)	102	+	1,59	1,16E-03	64,22
developmental process (GO:0032502)	161	+	1,36	1,54E-02	118,78
cellular process (GO:0009987)	600	+	1,16	9,23E-04	518,05
Unclassified (UNCLASSIFIED)	533	-	0,89	0,00E+00	601,42
response to stimulus (GO:0050896)	176	-	0,76	4,48E-03	231,97
neurological system process (GO:0050877)	70	-	0,67	4,22E-02	103,77
G-protein coupled receptor signaling pathway (GO:0007186)	22	-	0,44	1,68E-03	49,53
sensory perception (GO:0007600)	34	-	0,43	1,25E-06	78,72
sensory perception of chemical stimulus (GO:0007606)	9	-	< 0.2	4,80E-17	66,98
sensory perception of smell (GO:0007608)	6	-	< 0.2	3,82E-13	50,41
complement activation (GO:0006956)	1	-	< 0.2	3,51E-02	11,3
defense response to bacterium (GO:0042742)	1	-	< 0.2	3,32E-02	11,36
response to biotic stimulus (GO:0009607)	1	-	< 0.2	5,39E-03	13,31

B. KEGG 2016 term	Number of BA associated DMGs	P-value
Insulin secretion_Homo sapiens_hsa04911	16	0,0006
cGMP-PKG signaling pathway_Homo sapiens_hsa04022	24	0,0017
Calcium signaling pathway_Homo sapiens_hsa04020	25	0,0022
Insulin resistance_Homo sapiens_hsa04931	17	0,0032
Cholinergic synapse_Homo sapiens_hsa04725	16	0,0091
Type II diabetes mellitus_Homo sapiens_hsa04930	9	0,0089
Chagas disease (American trypanosomiasis)_Homo sapiens_hsa05142	15	0,0112
Inflammatory mediator regulation of TRP channels_Homo sapiens_hsa04750	14	0,0151
AGE-RAGE signaling pathway in diabetic complications_Homo sapiens_hsa04933	14	0,0193
GABAergic synapse_Homo sapiens_hsa04727	13	0,0145
Adrenergic signaling in cardiomyocytes_Homo sapiens_hsa04261	19	0,0158
Glycolysis / Gluconeogenesis_Homo sapiens_hsa00010	10	0,0280
Vascular smooth muscle contraction_Homo sapiens_hsa04270	15	0,0365
MAPK signaling pathway_Homo sapiens_hsa04010	27	0,0467

Supplemental 4. Adult CA-exposure leads to DMG involved in development and lipid metabolism. a/ Gene ontology analysis on differentially methylated genes in sperm cells of F0 mice fed 4 months with Control or 0.5%-CA diet. **b/** KEGG pathways analysis on differentially methylated genes in sperm cells of F0 mice fed 4 months with Control or 0.5%-CA diet.

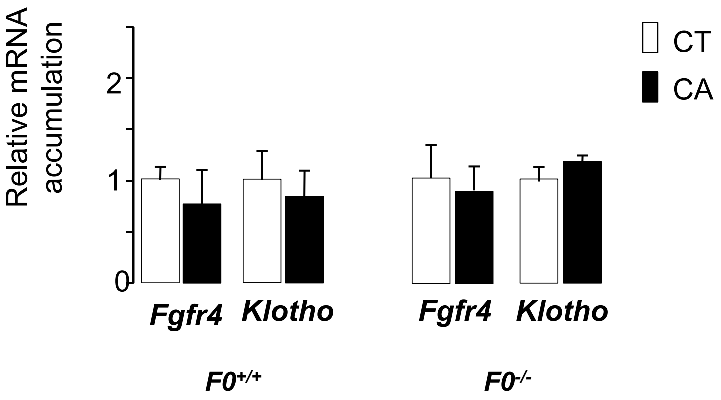
Relative mRNA accumulation
0
1
2
CT
CA
Fgfr4
Klotho
Fgfr4
Klotho
F0+/+
F0-/-

Supplemental 5. Liver mRNA accumulation of *Fgfr4* and *Klotho* normalized to *β-actin* mRNA levels in 15-days old $F1^{-CT;\ +/+}$, $F1^{CT;\ -/-}$, $F1^{CA;\ +/+}$ and $F1^{CA;\ -/-}$ mice.

In all panels data are expressed as means +/-SEM. $n = 6\text{-}15$ per group from 3 independent experiments. Statistical analysis: * $p < 0.05$ vs. control diet group.

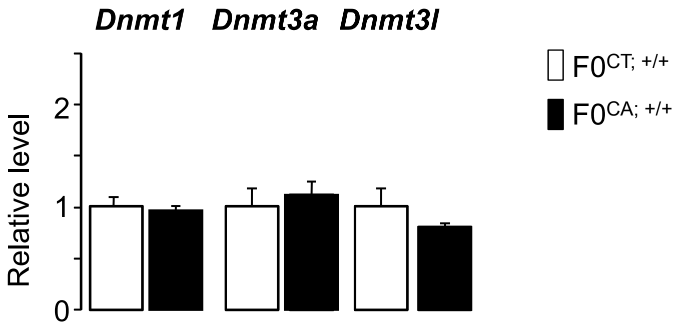
Dnmt1
Dnmt3a
Dnmt3l
Relative level
2
1
0
F0CT; +/+
F0CA; +/+
