# Supplementary material for: Multigenerational impacts of bile exposure are mediated by TGR5 signaling pathways
Source: Sci Rep. 2018 Nov 15;8:16875. doi: 10.1038/s41598-018-34863-0 (PMC6237852; doi:10.1038/s41598-018-34863-0)
Supplement: Supplementary file 3 — supplementary table 9 [file 41598_2018_34863_MOESM3_ESM.docx]

**Supplemental Table S9:** Sequences of primers used in this study.

G6Pasefw: CCGGATCTACCTTGCTGCYCACCTT

G6Paserev: TAGCAGGTAGAATCCAAGCGCGAAAC

Pepckfw: CCACAGCTGCTGCAGAACA

Pepckrev: GAAGGGTCGCATGGCAAA

Cyp46a1fw: AATGTGACTATGGGCGCTGG

Cyp46a1rev: CACGGGTGTCTGTCCATCTG

Fgfr4fw: CAGAGGCCTTTGGTATGGAT

Fgfr4rev: AGGTCTGCCAAATCCTTGTC

Klbfw: AGACTTTTGGAGACCGTGTCA

Klbrev: GAGAGCCAACCCTTCTGATGA

Kcnq1fw: TTTCTCTGCATGGTCCTTCC

Kcnq1rev: TTGAGCAAAGCACACTGAGG
